# Transsulfuration Pathway Thiols and Methylated Arginines: The Hunter Community Study

**DOI:** 10.1371/journal.pone.0054870

**Published:** 2013-01-24

**Authors:** Arduino A. Mangoni, Angelo Zinellu, Ciriaco Carru, John R. Attia, Mark McEvoy

**Affiliations:** 1 Division of Applied Medicine, University of Aberdeen, Aberdeen, United Kingdom; 2 Department of Biomedical Sciences, University of Sassari, Sassari, Italy; 3 Centre for Clinical Epidemiology and Biostatistics, University of Newcastle, Newcastle, NSW, Australia; Innsbruck Medical University, Austria

## Abstract

**Background:**

Serum homocysteine, when studied singly, has been reported to be positively associated both with the endogenous nitric oxide synthase inhibitor asymmetric dimethylarginine [ADMA, via inhibition of dimethylarginine dimethylaminohydrolase (DDAH) activity] and with symmetric dimethylarginine (SDMA). We investigated combined associations between transsulfuration pathway thiols, including homocysteine, and serum ADMA and SDMA concentrations at population level.

**Methods:**

Data on clinical and demographic characteristics, medication exposure, C-reactive protein, serum ADMA and SDMA (LC-MS/MS), and thiols (homocysteine, cysteine, taurine, glutamylcysteine, total glutathione, and cysteinylglycine; capillary electrophoresis) were collected from a sample of the Hunter Community Study on human ageing [n = 498, median age (IQR) = 64 (60–70) years].

**Results:**

Regression analysis showed that: a) age (*P* = 0.001), gender (*P* = 0.03), lower estimated glomerular filtration rate (eGFR, *P* = 0.08), body mass index (*P* = 0.008), treatment with beta-blockers (*P* = 0.03), homocysteine (*P* = 0.02), and glutamylcysteine (*P* = 0.003) were independently associated with higher ADMA concentrations; and b) age (*P* = 0.001), absence of diabetes (*P* = 0.001), lower body mass index (*P* = 0.01), lower eGFR (*P*<0.001), cysteine (*P* = 0.007), and glutamylcysteine (*P*<0.001) were independently associated with higher SDMA concentrations. No significant associations were observed between methylated arginines and either glutathione or taurine concentrations.

**Conclusions:**

After adjusting for clinical, demographic, biochemical, and pharmacological confounders the combined assessment of transsulfuration pathway thiols shows that glutamylcysteine has the strongest and positive independent associations with ADMA and SDMA. Whether this reflects a direct effect of glutamylcysteine on DDAH activity (for ADMA) and/or cationic amino acid transport requires further investigations.

## Introduction

The methylated forms of the amino acid L-arginine, asymmetric (ADMA) and symmetric (SDMA) dimethylarginine, are generated from the proteolysis of proteins containing methylated arginine residues [1], [2]. Both ADMA and, to a lesser extent, SDMA play an important role in cardiovascular homeostasis. ADMA is a potent endogenous inhibitor of endothelium nitric oxide synthase [1], [2]. Experimental and human studies have convincingly demonstrated that ADMA facilitates endothelial dysfunction, vascular damage, and the onset and progression of atherosclerosis and thrombosis [3]. Recent reports also suggest a potential, albeit indirect, role of SDMA in inhibiting nitric oxide synthesis and in favouring inflammation [4], [5]. Clinical studies conducted over the last 20 years have provided solid evidence that higher plasma ADMA and, more recently, SDMA concentrations independently predict adverse cardiovascular outcomes in several patient groups with different cardiovascular risk at baseline [6]–[11].

Cardiovascular disease biomarkers for clinical use should have several characteristics, i.e. easily measurable in the population, predictable relationship with cardiovascular risk, and modification by means of pharmacological and/or non-pharmacological interventions [12]. Currently, ADMA and SDMA possess the first two characteristics. Whilst future clinical studies are likely to address the issue of ADMA and SDMA modulation in relation to risk modification an important issue remains the identification of biological processes and pathways influencing methylated arginine synthesis and metabolism in humans.

**Figure 1:**
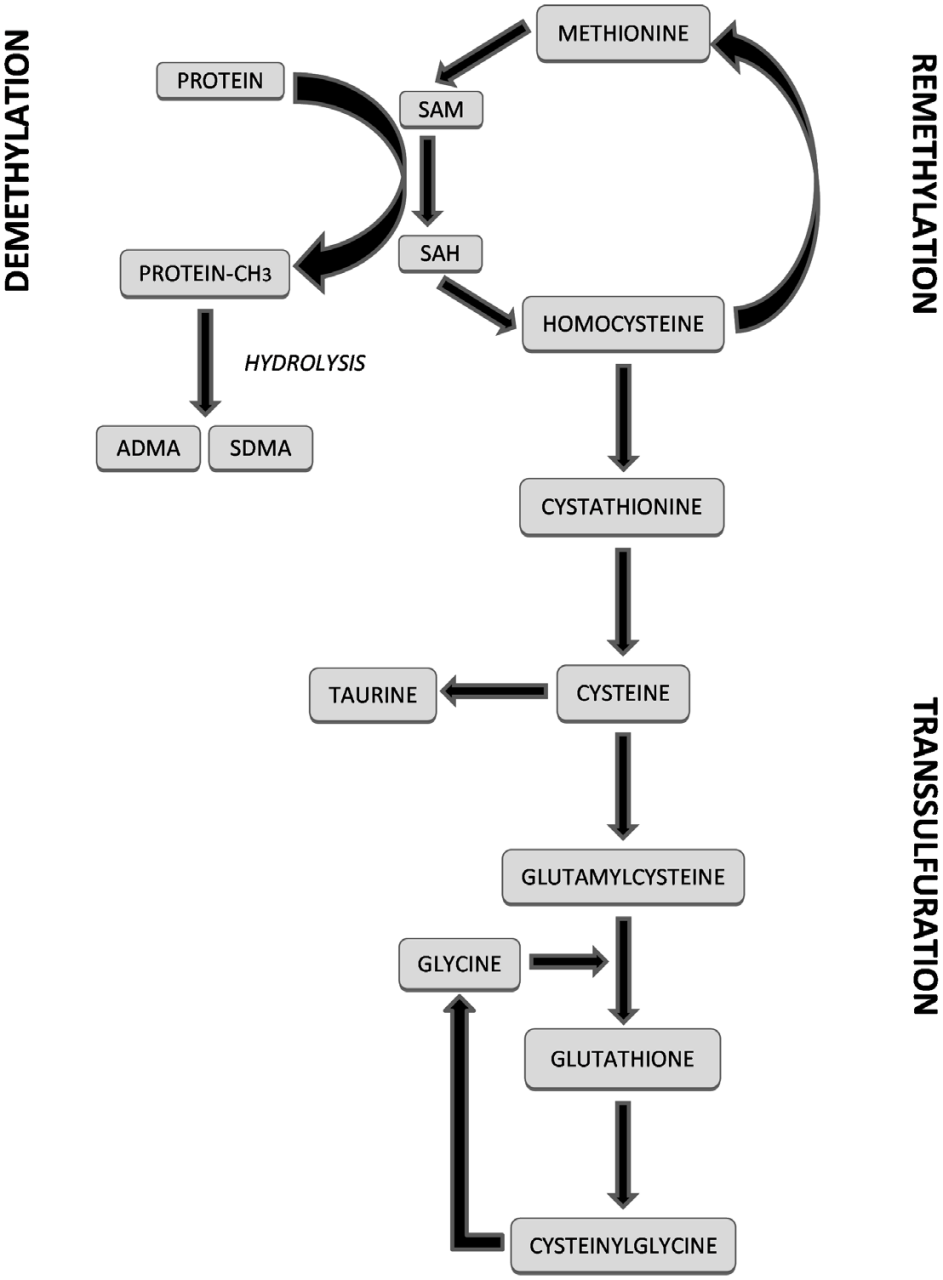
Relationships between the transsulfuration, demethylation, and remethylation pathways. ADMA: asymmetric dimethylarginine, SDMA: symmetric dimethylarginine, SAM: S-adenosylmethionine, SAH: S-adenosylhomocysteine.

The highly reactive sulphur-containing amino acid homocysteine has long been shown to exert negative effects on endothelial function by inhibiting nitric oxide synthesis [13]. Similarly to methylated arginines, several clinical studies have shown that higher homocysteine concentrations independently predict adverse cardiovascular outcomes and improve risk reclassification [13]–[15]. Notably, a number of human studies have shown positive associations between homocysteine, ADMA, and SDMA concentrations [7], [16]–[19]. The synthesis of ADMA and SDMA is catalyzed by a family of enzymes called protein-arginine-N-methyltransferases (PRMT). PRMT utilize S-adenosylmethionine, an intermediate in the methionine-homocysteine pathway, as a methyl donor [1]. After donating its methyl group, S-adenosylmethionine is transformed into S-adenosylhomocysteine, and then into homocysteine (Figure 1) [20]. *In vitro* studies have also demonstrated that homocysteine inhibits the activity of dimethylarginine dimethylaminohydrolase (DDAH), the enzyme responsible for ADMA metabolism [21], [22]. These findings suggest a biological and pathophysiological interplay between homocysteine, methylated arginines, and cardiovascular disease [23].

Homocysteine is the initial step of the transsulfuration pathway [20]. This biochemical pathway leads to the synthesis of important cellular and homeostatic thiols such as cysteine, taurine, and the natural antioxidant glutathione (Figure 1) [24]–[26]. Little knowledge is currently available on whether there are associations between transsulfuration pathway thiols and methylated arginines [27]. Ideally, human studies investigating these associations should account for a number of clinical, demographic, biochemical, and pharmacological confounders affecting these pathways [1], [2], [13], [28], [29]. We addressed this issue by examining the combined associations between transsulfuration pathway thiols and serum concentrations of ADMA and SDMA at population level, in an established epidemiological cohort of human ageing.

## Methods

### Population

The Hunter Community Study (HCS), a collaboration between the University of Newcastle’s School of Medicine and Public Health and the Hunter New England Area Health Service, is a population-based cohort study to assess the impact of clinical, genetic, biochemical, socioeconomic, and behavioural factors on human ageing [30]. Participants, a cohort of community-dwelling subjects aged 55–85 years residing in Newcastle (New South Wales, Australia), were randomly selected from the electoral roll and contacted between December 2004 and December 2007. Invitation letters were sent to 9,784 individuals. Of the 7,575 subjects for whom a response was received, 258 were ineligible (148 did not speak English, 92 were deceased, and 18 had moved to an aged-care facility), 3,440 refused, and 3,877 initially agreed to participate. Of these, a total of 3,253 actually participated (response rate 44.5%).

After informed, written consent was obtained, subjects were asked to complete two self-report questionnaires and to return these when they attended the HCS data collection centre, during which time a series of clinical and biochemical measures was obtained. Clinical assessment included a full physical examination and measurement of blood pressure, heart rate, body mass index, and waist-to-hip ratio. Routine haematological and biochemical parameters included full blood count, C-reactive protein (CRP), fasting lipids, liver and renal function, and fasting blood glucose. Additional samples were cryopreserved at −86°C and −196 °C. Consent to link personal information obtained during the study to data from Medicare Australia and local health databases was also sought.

After the clinical assessment a further package of three self-reporting questionnaires to be returned by reply-paid post was given to participants to complete at home. The questionnaires provided details on demographic and socioeconomic characteristics, nutritional assessment, medical and surgical history, medication exposure, tobacco use, and alcohol consumption. Full details of the data collected are described elsewhere [30].

The sample for this investigation (n = 500) was derived from the initial cohort by simple random sampling. Of the 500 subjects randomly selected there were complete exposure and outcome data for 498 subjects. No a priori sample size was determined, however assuming that at least 10–15 subjects are needed for each independent variable included in the multivariate analysis the sample size was more than sufficient to accommodate the number of co-variables examined in this investigation (see Statistical analysis paragraph). A comparison of this sample with the entire cohort showed no significant difference for a range of clinical, biochemical, socioeconomic, and behavioural factors (data not shown). The HCS was performed according to the Declaration of Helsinki. All procedures were approved by the local ethics committee.

### Biochemical Measurements

Blood was collected in EDTA tubes and centrifuged at 4° and 3000 g for 10 minutes to separate plasma, which was stored for three years at −80°C before analysis. L-arginine, ADMA, and SDMA were measured in 0.1 mL serum by hydrophilic-interaction liquid chromatography and isotope dilution tandem mass spectrometry [31]. The intra and inter-assay coefficients of variation (CV) for L-arginine, ADMA, and SDMA were all <15%. Serum concentrations of the transsulfuration pathway thiols homocysteine, cysteine, cysteinylglycine, glutamylcysteine, glutathione, and taurine were measured by laser-induced fluorescence capillary electrophoresis on 0.05 mL serum for taurine and 0.2 mL for the other thiols [32], [33]. A five-point calibration curve was used to measure analyte concentrations. Only for taurine was homocysteic acid used as internal standard [33]. The minimum detectable concentration for all analytes was between 200 and 300 pmol/L, with mean recovery between 98% and 102%. A good reproducibility of intra-assay (CV <3.5%) and inter-assay (CV <6.4%) tests was obtained [32], [33]. High-sensitivity CRP was measured in serum by latex-enhanced immunoturbidimetry. Estimated glomerular filtration rate (eGFR) was calculated using the Modification of Diet in Renal Disease formula [34].

### Statistical Analysis

Results are expressed as means ± SD, medians and interquartile ranges, or frequencies as appropriate. Variables were tested for normal distribution by using the Kolmogorov-Smirnov test. Univariate associations between clinical and demographic variables, thiols, ADMA, and SDMA were assessed by Spearman’s rank correlation coefficient, two-way ANOVA, and Mann-Whitney U test. Non-normally distributed variables were log transformed. Variables showing associations with either ADMA or SDMA (*P*<0.2) were entered in linear stepwise regression analysis to identify factors independently associated with methylated arginines. Only log-transformed variables were tested in a single analysis. Multicollinearity was tested by measuring the tolerance and the variance inflation factor values for each analysis. A total of 31 variables were identified *a priori* to be potentially associated with the outcomes of interest: age, gender, body mass index, smoking, alcohol consumption, history of hypertension, hypercholesterolaemia, rheumatoid arthritis, myocardial infarction, stroke, diabetes, fasting glucose, total cholesterol, HDL and LDL cholesterol, triglycerides, eGFR, CRP, homocysteine, cysteine, taurine, glutamylcysteine, glutathione, cysteinylglycine, and use of antiplatelet drugs, beta-blockers, angiotensin converting enzyme inhibitors, statins, diuretics, calcium channel blockers, and antidiabetic drugs. Considering at least 15 patients per each confounding variable a minimum sample size of 465 patients was required for regression analyses [35]. Analyses were performed using IBM SPSS Statistics 19.0 for Windows (SPSS Inc, Chicago, IL, USA). A two-sided *P*<0.05 indicated statistical significance. The latter was adjusted, *P*<0.01, in regression analysis to account for multiple comparisons.

## Results

Clinical, demographic, and biochemical characteristics and medication use of the study population are described in Table 1. Folic acid and vitamin B_12_ supplements were used in a relatively small proportion of participants, 1.8% and 1.4%, respectively. There were no significant differences in homocysteine and cysteine concentrations between those with vs. without folic acid [9.1 (5.9–9.9) vs. 9.1 (7.9–11.0) µmol/L, *P* = 0.32; 178.4±29.6 vs. 190.3±34.1 µmol/L, *P* = 0.30) and between those with vs. without vitamin B_12_ [8.3 (7.7–9.1) vs. 9.1 (7.9–10.9) µmol/L, *P* = 0.26; 172.0±23.6 vs. 190.4±34.1 µmol/L, *P* = 0.16).

**Table 1:** Clinical, demographic, biochemical characteristics and medication use.

Variable	Study population (n = 498)
Age [years, median (IQR)]	64 (60–70)
Females (%)	49.4
Current smoker (%)	6.6
Current alcohol use (%)	69.7
Body mass index [Kg/m^2^, median (IQR)]	28.0 (25.7–31.2)
Systolic blood pressure (mmHg, mean±SD)	137±18
Diastolic blood pressure (mmHg, mean±SD)	80±10
Heart rate (b/min, mean±SD)	66±11
Hypertension (%)	49.3
Rheumatoid arthritis (%)	5.5
Diabetes (%)	10.8
Hypercholesterolaemia (%)	40.8
Myocardial infarction (%)	5.7
Stroke (%)	2.6
Antiplatelet drugs (%)	2.5
Beta-blockers (%)	21.4
Angiotensin converting enzyme inhibitors (%)	47.4
Calcium-channel blockers (%)	34.9
Statins (%)	13.0
Diuretics (%)	9.7
Antidiabetic drugs (%)	6.5
Folic acid supplements (%)	1.8
Vitamin B_12_ supplements (%)	1.4
Fasting serum glucose [mmol/L, median (IQR)]	4.8 (4.4–5.4)
Total cholesterol [mmol/L, median (IQR)]	4.9 (4.3–5.8)
HDL-cholesterol [mmol/L, median (IQR)]	1.3 (1.1–1.5)
LDL-cholesterol (mmol/L, mean±SD)	3.1±0.9
Triglycerides [mmol/L, median (IQR)]	1.1 (0.8–1.6)
eGFR^a^ (mL/min, mean±SD)	79±16
C-reactive protein [mg/L, median (IQR)]	2.0 (1.2–3.7)
Homocysteine [µmol/L, median (IQR)]	9.1 (7.9–10.8)
Cysteine (µmol/L, mean±SD)	187.8±37.6
Taurine [µmol/L, median (IQR)]	63.3 (52.3–89.7)
Glutamylcysteine (µmol/L, mean±SD)	4.4±1.2
Glutathione [µmol/L, median (IQR)]	3.9 (3.0–5.3)
Cysteinylglycine [µmol/L, median (IQR)]	29.1 (25.4–33.4)
L-arginine (µmol/L, mean±SD)	55.3±18.7
Asymmetric dimethylarginine (µmol/L, mean±SD)	0.54±0.08
Symmetric dimethylarginine [µmol/L, median (IQR)]	0.69 (0.61–0.82)

a calculated using the Modification of Diet in Renal Disease formula.

### ADMA

Age, body mass index, lower total and HDL cholesterol, lower eGFR, CRP, and higher concentrations of all thiols were associated (*P*<0.2) with higher ADMA concentrations (Table 2 and Figure S1). Associations were also found with gender (female: 0.55±0.07 vs. male: 0.54±0.08 µmol/L, *P* = 0.15), regular alcohol consumption (no: 0.55±0.08 vs. yes: 0.54±0.07 µmol/L, *P* = 0.05), hypertension (no: 0.53±0.07 vs. yes: 0.56±0.08 µmol/L, *P* = 0.001), myocardial infarction (no: 0.54±0.08 vs. yes: 0.59±0.07 µmol/L, *P* = 0.004), stroke (no: 0.54±0.08 vs. yes: 0.58±0.07 µmol/L, *P* = 0.12), antiplatelet drugs (no: 0.55±0.08 vs. yes: 0.58±0.08 µmol/L, *P* = 0.16), beta-blockers (no: 0.54±0.07 vs. yes: 0.58±0.08 µmol/L, *P*<0.001), angiotensin converting enzyme inhibitors (no: 0.54±0.07 vs. yes: 0.56±0.08 µmol/L, *P* = 0.04), statins (no: 0.54±0.08 vs. yes: 0.56±0.08 µmol/L, *P* = 0.15), and diuretics (no: 0.54±0.07 vs. yes: 0.59±0.09 µmol/L, *P*<0.001).

**Table 2:** Correlations between ADMA and SDMA concentrations, clinical and demographic factors, and biochemical variables.

Variable	ADMA	SDMA
Age	r = +0.24, *P*<0.00001	r = +0.31, *P*<0.00001
Body mass index	r = +0.10, *P* = 0.02	r = −0.18, *P* = 0.00007
Fasting serum glucose	r = −0.003, *P* = 0.95	r = −0.16, *P* = 0.001
Total cholesterol	r = −0.11, *P* = 0.01	r = −0.13, *P* = 0.004
HDL-cholesterol	r = −0.05, *P* = 0.24	r = +0.03, *P* = 0.48
LDL-cholesterol	r = −0.09, *P* = 0.06	r = −0.06, *P* = 0.17
Triglycerides	r = +0.01, *P* = 0.79	r = −0.20, *P* = 0.00001
eGFR	r = −0.24, *P*<0.00001	r = −0.47, *P*<0.00001
C-reactive protein	r = +0.08, *P* = 0.09	r = −0.06, *P* = 0.16
Homocysteine	r = +0.24, *P*<0.00001	r = +0.24, *P*<0.00001
Cysteine	r = +0.21, *P* = 0.00001	r = +0.27, *P*<0.00001
Taurine	r = +0.10, *P = *0.03	r = +0.01, *P* = 0.79
Glutamylcysteine	r = +0.30, *P*<0.00001	r = +0.35, *P*<0.00001
Glutathione	r = +0.10, *P* = 0.03	r = +0.16, *P* = 0.0004
Cysteinylglycine	r = +0.14, *P* = 0.002	r = +0.12, *P* = 0.007

On regression analysis age, female gender, lower eGFR, body mass index, treatment with beta-blockers, and the thiols homocysteine and glutamylcysteine were independently associated with higher serum ADMA concentrations. Glutamylcysteine showed stronger associations with ADMA concentrations vs. homocysteine (Table 3).

**Table 3:** Forward stepwise regression of serum ADMA concentrations.

Variables	B coefficient (95% CI)	P-value
Log age	0.130 (0.054 to 0.205)	0.001
Gender (0 = female, 1 = male)	−0.017 (−0.033 to −0.002)	0.03*
Log body mass index	0.066 (0.018 to 0.114)	0.008
eGFR	−0.00047 (−0.00101 to 0.00006)	0.08*
Beta blockers (0 = no, 1 = yes)	0.022 (0.002 to 0.041)	0.03*
Log homocysteine	0.035 (0.005 to 0.064)	0.02*
Glutamylcysteine	0.011 (0.004 to 0.018)	0.003

Variables entered in the model: age, gender, body mass index, current alcohol use, hypertension, myocardial infarction, stroke, eGFR, C-reactive protein, total cholesterol, LDL-cholesterol, antiplatelet drugs, angiotensin converting enzyme inhibitors, statins, diuretics, beta-blockers, homocysteine, glutamylcysteine, cysteinylglycine, cysteine, glutathione, taurine.

* not significant after adjusting level of significance (*P*<0.01).

### SDMA

Age, lower body mass index, lower serum glucose, lower total and LDL cholesterol, lower triglycerides, lower CRP and eGFR, and higher concentrations of all thiols except taurine were associated (*P*<0.2) with higher SDMA concentrations (Table 2 and Figure S2). Associations were also found with regular alcohol consumption (no: 0.75±0.20 vs. yes: 0.72±0.17 µmol/L, *P* = 0.04), diabetes (no: 0.73±0.18 vs. yes: 0.68±0.17 µmol/L, *P* = 0.04), myocardial infarction (no: 0.73±0.18 vs. yes: 0.79±0.20 µmol/L, *P* = 0.05), stroke (no: 0.72±0.18 vs. yes: 0.87±0.20 µmol/L, *P* = 0.003), antiplatelet drugs (no: 0.73±0.18 vs. yes: 0.84±0.13 µmol/L, *P* = 0.08), angiotensin converting enzyme inhibitors (no: 0.71±0.16 vs. yes: 0.76±0.21 µmol/L, *P* = 0.007), beta-blockers (no: 0.72±0.18 vs. yes: 0.78±0.20 µmol/L, *P* = 0.008), and diuretics (no: 0.72±0.17 vs. yes: 0.82±0.27 µmol/L, *P* = 0.001).

On regression analysis age, absence of diabetes, lower body mass index, lower eGFR, and the thiols cysteine and glutamylcysteine were independently associated with higher serum SDMA concentrations (Table 4).

**Table 4:** Forward stepwise regression of serum SDMA concentrations.

Variables	B coefficient (95% CI)	P-value
Log age	0.142 (0.057 to 0.227)	0.001
Log body mass index	−0.070 (−0.124 to −0.016)	0.012*
Diabetes	−0.043 (−0.069 to −0.017)	0.001
eGFR	−0.00260 (−0.00317 to −0.00203)	<0.00001
Cysteine	0.00034 (0.00009 to 0.00059)	0.007
Glutamylcysteine	0.015 (0.007 to 0.023)	0.00009

Variables entered in the model: age, body mass index, current alcohol use, myocardial infarction, stroke, eGFR, C-reactive protein, total cholesterol, LDL-cholesterol, antiplatelet drugs, angiotensin converting enzyme inhibitors, diuretics, beta-blockers, homocysteine, glutamylcysteine, cysteinylglycine, cysteine, glutathione.

* not significant after adjusting level of significance (*P*<0.01).

## Discussion

Three transsulfuration pathway thiols showed significant, independent, and positive associations with serum concentrations of methylated arginines in an established epidemiological cohort of human ageing. After adjusting for clinical, demographic, biochemical, and pharmacological confounders, homocysteine and glutamylcysteine were both associated with higher ADMA concentrations whereas cysteine and glutamylcysteine were both associated with higher SDMA concentrations. Of note, no independent associations were observed with the antioxidant thiols glutathione and taurine.

The transsulfuration pathway regulates important physiological and homeostatic processes, including detoxification of xenobiotics or their metabolites, maintenance of intracellular redox balance and thiol status of proteins, and ensuring cysteine storage within the γ-glutamyl cycle [24]–[26], [36]. Our results confirm previous reports demonstrating associations between serum homocysteine, the first step of the transsulfuration pathway, and ADMA concentrations [7], [16]–[19]. Possible mechanisms for the increased ADMA concentrations include the involvement of the methionine-homocysteine pathway in the biosynthesis of ADMA and the homocysteine-mediated inhibition of ADMA metabolism by the enzyme DDAH [21], [22]. This might explain the co-existence of elevated homocysteine and ADMA concentrations, vascular damage, and adverse outcomes reported in several studies [7], [37]–[39]. By contrast, no independent associations were observed between homocysteine and SDMA concentrations. Significant independent associations between homocysteine and SDMA have been previously reported [7], [19], [40]. Although clinical and demographic factors were considered in these studies, a possible reason for the different results in our study is the combined assessment of several transsulfuration pathway thiols, some showing stronger independent associations with SDMA.

The thiol glutamylcysteine showed the strongest independent associations with higher ADMA and SDMA concentrations. Glutamylcysteine, the immediate precursor of glutathione, is synthesised by the enzyme glutamylcysteine synthetase. The catalytic activity of glutamylcysteine synthetase depends on the availability of cysteine and is inhibited by glutathione [41]. There is increasing evidence that glutamylcysteine plays an important role in modulating oxidative stress and cardiovascular risk. Nakamura et al have recently reported a significant dose-dependent reduction in markers of oxidative stress in human endothelial cells exposed to glutamylcysteine [42]. Although intracellular concentrations might differ from serum glutamylcysteine concentrations the effects on oxidative stress were observed at concentrations, i.e. ≥50 µmol/L, significantly higher than those reported in our study. Moreover, polymorphisms of the enzyme glutamylcysteine synthetase are associated with reduced endothelial function and increased risk of myocardial infarction [43]. There are at least two possible mechanisms by which glutamylcysteine might modulate ADMA and SDMA concentrations: 1) a direct inhibitory effect of glutamylcysteine on DDAH expression and/or activity, with a consequent increase in ADMA concentrations, similarly to that reported with homocysteine [21], [22]; 2) the role of glutamylcysteine as part of the γ-glutamyl cycle. The latter has been shown to modulate the trans-membrane transport of several amino acids, including arginine [44]. A similar phenomenon might involve the methylated forms ADMA and SDMA. Further *in vitro* research is necessary to corroborate these findings and to provide mechanistic insights.

An independent and positive association, not previously reported, was also demonstrated between the thiol cysteine and SDMA concentrations. Whether this reflects the role of cysteine in the γ-glutamyl cycle, similarly to glutamylcysteine, and potentially in SDMA transport requires further studies. Glutathione and taurine have been shown to modulate DDAH activity *in vitro*. It has been speculated that the effects on DDAH activity are largely mediated by the antioxidant effects of these thiols [45]–[47]. However, no associations were observed between glutathione, taurine, and methylated arginines.

The association between several clinical and demographic characteristics, e.g. age, renal function, and body mass index, and methylated arginine concentrations is in line with previous reports [1], [2], [48]. Although the independent negative association between body mass index and serum SDMA concentrations is apparently counterintuitive, our results are in line with a recently published study. Schwedhelm et al observed negative associations between body mass index and SDMA concentrations both in univariate (r = −0.13, *P*<0.001) and regression analyses (B coefficient −0.0031, *P*<0.01) [19]. Two further studies have demonstrated independent negative associations between SDMA and insulin resistance, commonly associated with higher body mass index [49], [50]. Similarly, we observed a negative association between fasting serum glucose and SDMA (Table 2). It has been speculated that insulin resistance might selectively promote cellular uptake of SDMA through increased expression of the y+ transporter [51]. However, further research is warranted to clarify this issue.

Although female gender was associated with higher ADMA concentrations in our study, previous reports on the impact of gender on ADMA have provided conflicting results. This might be secondary to differences in study population, e.g. age, and statistical approach [52]–[54]. The use of beta-blockers as a class was associated with higher ADMA concentrations. Previous studies have shown contrasting effects of beta-blockers on ADMA concentrations. It is possible that the discrepancy in the results of these reports depends, at least partly, on the use of specific beta-blockers [55]–[57]. Of note, a history of diabetes was independently associated with lower SDMA concentrations. As diabetes is frequently associated with the presence of kidney disease, hence a reduced SDMA clearance, this finding is also apparently counterintuitive. However, a recent study has demonstrated that SDMA concentrations in patients with type 2 diabetes depend on glycaemic control. Can et al observed that patients with relatively poor glycaemic control had lower SDMA concentrations vs. patients with good control and healthy subjects [58]. The presence of a negative correlation between SDMA and both HbA1c and fructosamine suggests an interaction between protein methylation and glucose homeostasis [58]. Moreover, as previously discussed, there is evidence that SDMA is inversely associated with insulin resistance [49], [50]. In line with these findings our study demonstrated negative correlations between fasting serum glucose concentrations and SDMA concentrations (Table 2).

A limitation of our study is its cross-sectional nature, which does not allow the assessment of cause-effect relationship between transsulfuration thiols and methylated arginines. Moreover, similarly to most population studies on methylated arginines, the measurement of transsulfuration thiols, ADMA, and SDMA from blood does not necessarily reflect intracellular concentrations of these compounds. Another important issue is the risk of falsely positive associations due to multiple comparisons in regression analysis. Adjustment of the level of significance according to established approaches, e.g. Bonferroni correction, might be too conservative in this context [59]. Although the level of significance was lowered to *P*<0.01 in regression analysis, the possibility of data over-interpretation cannot be ruled out. On the other hand, strengths of this study are the combined assessment of transsulfuration thiols and the adjustment for several clinical, demographic, biochemical, and pharmacologic confounders in regression analysis.

### Conclusions

This study has shown significant associations between three transsulfuration pathway thiols, particularly glutamylcysteine, and methylated arginines at population level. Further *in vitro* studies are necessary to clarify the mechanism responsible for these associations, e.g. direct effects on ADMA metabolism and/or interactions between the γ-glutamyl cycle and amino acid transmembrane transport.

## Supporting Information

Figure S1 **Scatter plots between individual serum thiols and ADMA concentrations.**
(PPTX)

Figure S2 **Scatter plots between individual serum thiols and SDMA concentrations.**
(PPTX)

## References

[1] Vallance P, Leiper J (2004) Cardiovascular biology of the asymmetric dimethylarginine:dimethylarginine dimethylaminohydrolase pathway. Arterioscler Thromb Vasc Biol 24: 1023–1030. 15105281 10.1161/01.ATV.0000128897.54893.26

[2] Mangoni AA (2009) The emerging role of symmetric dimethylarginine in vascular disease. Adv Clin Chem 48: 73–94. 19803415 10.1016/s0065-2423(09)48003-x

[3] Boger RH (2003) The emerging role of asymmetric dimethylarginine as a novel cardiovascular risk factor. Cardiovasc Res 59: 824–833. 14553822 10.1016/s0008-6363(03)00500-5

[4] Bode-Boger SM, Scalera F, Kielstein JT, Martens-Lobenhoffer J, Breithardt G, et al (2006) Symmetrical dimethylarginine: a new combined parameter for renal function and extent of coronary artery disease. J Am Soc Nephrol 17: 1128–1134. 16481412 10.1681/ASN.2005101119

[5] Schepers E, Barreto DV, Liabeuf S, Glorieux G, Eloot S, et al (2011) Symmetric dimethylarginine as a proinflammatory agent in chronic kidney disease. Clin J Am Soc Nephrol 6: 2374–2383. 21817129 10.2215/CJN.01720211 PMC3359555

[6] Boger RH, Endres HG, Schwedhelm E, Darius H, Atzler D, et al (2011) Asymmetric dimethylarginine as an independent risk marker for mortality in ambulatory patients with peripheral arterial disease. J Intern Med 269: 349–361. 21175900 10.1111/j.1365-2796.2010.02322.x

[7] Meinitzer A, Kielstein JT, Pilz S, Drechsler C, Ritz E, et al (2011) Symmetrical and asymmetrical dimethylarginine as predictors for mortality in patients referred for coronary angiography: the Ludwigshafen Risk and Cardiovascular Health study. Clin Chem 57: 112–121. 21036946 10.1373/clinchem.2010.150854

[8] Cavusoglu E, Ruwende C, Chopra V, Poludasu S, Yanamadala S, et al (2010) Relation of baseline plasma ADMA levels to cardiovascular morbidity and mortality at two years in men with diabetes mellitus referred for coronary angiography. Atherosclerosis 210: 226–231. 19944421 10.1016/j.atherosclerosis.2009.10.034

[9] Schulze F, Carter AM, Schwedhelm E, Ajjan R, Maas R, et al (2010) Symmetric dimethylarginine predicts all-cause mortality following ischemic stroke. Atherosclerosis 208: 518–523. 19700158 10.1016/j.atherosclerosis.2009.06.039

[10] Zoccali C, Bode-Boger S, Mallamaci F, Benedetto F, Tripepi G, et al (2001) Plasma concentration of asymmetrical dimethylarginine and mortality in patients with end-stage renal disease: a prospective study. Lancet 358: 2113–2117. 11784625 10.1016/s0140-6736(01)07217-8

[11] Boger RH, Sullivan LM, Schwedhelm E, Wang TJ, Maas R, et al (2009) Plasma asymmetric dimethylarginine and incidence of cardiovascular disease and death in the community. Circulation 119: 1592–1600. 19289633 10.1161/CIRCULATIONAHA.108.838268 PMC2742491

[12] Mayeux R (2004) Biomarkers: potential uses and limitations. NeuroRx 1: 182–188. 15717018 10.1602/neurorx.1.2.182 PMC534923

[13] Mangoni AA, Jackson SH (2002) Homocysteine and cardiovascular disease: current evidence and future prospects. Am J Med 112: 556–565. 12015248 10.1016/s0002-9343(02)01021-5

[14] Mangoni AA, Woodman RJ (2011) Homocysteine and cardiovascular risk an old foe creeps back. J Am Coll Cardiol 58: 1034–1035. 21867838 10.1016/j.jacc.2011.05.029

[15] Veeranna V, Zalawadiya SK, Niraj A, Pradhan J, Ference B, et al (2011) Homocysteine and reclassification of cardiovascular disease risk. J Am Coll Cardiol 58: 1025–1033. 21867837 10.1016/j.jacc.2011.05.028

[16] Boger RH, Lentz SR, Bode-Boger SM, Knapp HR, Haynes WG (2001) Elevation of asymmetrical dimethylarginine may mediate endothelial dysfunction during experimental hyperhomocyst(e)inaemia in humans. Clin Sci (Lond) 100: 161–167. 11171285

[17] Yoo JH, Lee SC (2001) Elevated levels of plasma homocyst(e)ine and asymmetric dimethylarginine in elderly patients with stroke. Atherosclerosis 158: 425–430. 11583722 10.1016/s0021-9150(01)00444-0

[18] Krzyzanowska K, Mittermayer F, Krugluger W, Schnack C, Hofer M, et al (2006) Asymmetric dimethylarginine is associated with macrovascular disease and total homocysteine in patients with type 2 diabetes. Atherosclerosis 189: 236–240. 16414052 10.1016/j.atherosclerosis.2005.12.007

[19] Schwedhelm E, Xanthakis V, Maas R, Sullivan LM, Atzler D, et al (2011) Plasma symmetric dimethylarginine reference limits from the Framingham offspring cohort. Clin Chem Lab Med 49: 1907–1910. 21864208 10.1515/CCLM.2011.679 PMC3235736

[20] Finkelstein JD, Martin JJ (2000) Homocysteine. Int J Biochem Cell Biol 32: 385–389. 10762063 10.1016/s1357-2725(99)00138-7

[21] Stuhlinger MC, Tsao PS, Her JH, Kimoto M, Balint RF, et al (2001) Homocysteine impairs the nitric oxide synthase pathway: role of asymmetric dimethylarginine. Circulation 104: 2569–2575. 11714652 10.1161/hc4601.098514

[22] Hong L, Fast W (2007) Inhibition of human dimethylarginine dimethylaminohydrolase-1 by S-nitroso-L-homocysteine and hydrogen peroxide. Analysis, quantification, and implications for hyperhomocysteinemia. J Biol Chem 282: 34684–34692. 17895252 10.1074/jbc.M707231200

[23] Dayal S, Lentz SR (2005) ADMA and hyperhomocysteinemia. Vasc Med 10 Suppl 1S27–S33. 16444866 10.1191/1358863x05vm599oa

[24] Go YM, Jones DP (2011) Cysteine/cystine redox signaling in cardiovascular disease. Free Radic Biol Med 50: 495–509. 21130865 10.1016/j.freeradbiomed.2010.11.029 PMC3040416

[25] Yamori Y, Taguchi T, Hamada A, Kunimasa K, Mori H, et al (2010) Taurine in health and diseases: consistent evidence from experimental and epidemiological studies. J Biomed Sci 17 Suppl 1S6. 20804626 10.1186/1423-0127-17-S1-S6 PMC2994368

[26] Ballatori N, Krance SM, Notenboom S, Shi S, Tieu K, et al (2009) Glutathione dysregulation and the etiology and progression of human diseases. Biol Chem 390: 191–214. 19166318 10.1515/BC.2009.033 PMC2756154

[27] Zinellu A, Sotgia S, Porcu P, Casu MA, Bivona G, et al (2011) Carotid restenosis is associated with plasma ADMA concentrations in carotid endarterectomy patients. Clin Chem Lab Med 49: 897–901. 21288172 10.1515/CCLM.2011.121

[28] Varela-Moreiras G (2001) Nutritional regulation of homocysteine: effects of drugs. Biomed Pharmacother 55: 448–453. 11686578 10.1016/s0753-3322(01)00126-3

[29] Dickinson DA, Forman HJ (2002) Cellular glutathione and thiols metabolism. Biochem Pharmacol 64: 1019–1026. 12213601 10.1016/s0006-2952(02)01172-3

[30] McEvoy M, Smith W, D’Este C, Duke J, Peel R, et al (2010) Cohort profile: The Hunter Community Study. Int J Epidemiol 39: 1452–1463. 20056765 10.1093/ije/dyp343

[31] Schwedhelm E, Tan-Andresen J, Maas R, Riederer U, Schulze F, et al (2005) Liquid chromatography-tandem mass spectrometry method for the analysis of asymmetric dimethylarginine in human plasma. Clin Chem 51: 1268–1271. 15976109 10.1373/clinchem.2004.046037

[32] Zinellu A, Carru C, Galistu F, Usai MF, Pes GM, et al (2003) N-methyl-D-glucamine improves the laser-induced fluorescence capillary electrophoresis performance in the total plasma thiols measurement. Electrophoresis 24: 2796–2804. 12929176 10.1002/elps.200305570

[33] Zinellu A, Sotgia S, Scanu B, Chessa R, Gaspa L, et al (2009) Taurine determination by capillary electrophoresis with laser-induced fluorescence detection: from clinical field to quality food applications. Amino Acids 36: 35–41. 18193477 10.1007/s00726-007-0022-5

[34] Levey AS, Bosch JP, Lewis JB, Greene T, Rogers N, et al (1999) A more accurate method to estimate glomerular filtration rate from serum creatinine: a new prediction equation. Modification of Diet in Renal Disease Study Group. Ann Intern Med 130: 461–470. 10075613 10.7326/0003-4819-130-6-199903160-00002

[35] Miller DE, Kunce JT (1973) Prediction and statistical overkill revisited. In: Measurement and Evaluation in Guidance. Association for Measurement and Evaluation in Guidance. 157–163.

[36] Meister A (1974) Glutathione, metabolism and function via the gamma-glutamyl cycle. Life Sci 15: 177–190. 4620960 10.1016/0024-3205(74)90206-9

[37] Mamatha SN, Nagaraja D, Philip M, Christopher R (2011) Asymmetric dimethylarginine as a risk marker for early-onset ischemic stroke in Indian population. Clin Chim Acta 412: 139–142. 20883678 10.1016/j.cca.2010.09.026

[38] Perna M, Roman MJ, Alpert DR, Crow MK, Lockshin MD, et al (2010) Relationship of asymmetric dimethylarginine and homocysteine to vascular aging in systemic lupus erythematosus patients. Arthritis Rheum 62: 1718–1722. 20155836 10.1002/art.27392

[39] Mao D, Che J, Li K, Han S, Yue Q, et al (2010) Association of homocysteine, asymmetric dimethylarginine, and nitric oxide with preeclampsia. Arch Gynecol Obstet 282: 371–375. 19806356 10.1007/s00404-009-1234-6

[40] Korandji C, Zeller M, Guilland JC, Vergely C, Sicard P, et al (2007) Asymmetric dimethylarginine (ADMA) and hyperhomocysteinemia in patients with acute myocardial infarction. Clin Biochem 40: 66–72. 17020756 10.1016/j.clinbiochem.2006.08.004

[41] Soltaninassab SR, Sekhar KR, Meredith MJ, Freeman ML (2000) Multi-faceted regulation of gamma-glutamylcysteine synthetase. J Cell Physiol 182: 163–170. 10623879 10.1002/(SICI)1097-4652(200002)182:2<163::AID-JCP4>3.0.CO;2-1

[42] Nakamura YK, Dubick MA, Omaye ST (2012) gamma-Glutamylcysteine inhibits oxidative stress in human endothelial cells. Life Sci 90: 116–121. 22075492 10.1016/j.lfs.2011.10.016

[43] Koide S, Kugiyama K, Sugiyama S, Nakamura S, Fukushima H, et al (2003) Association of polymorphism in glutamate-cysteine ligase catalytic subunit gene with coronary vasomotor dysfunction and myocardial infarction. J Am Coll Cardiol 41: 539–545. 12598062 10.1016/s0735-1097(02)02866-8

[44] Orlowski M, Meister A (1970) The gamma-glutamyl cycle: a possible transport system for amino acids. Proc Natl Acad Sci U S A 67: 1248–1255. 5274454 10.1073/pnas.67.3.1248 PMC283344

[45] Pope AJ, Druhan L, Guzman JE, Forbes SP, Murugesan V, et al (2007) Role of DDAH-1 in lipid peroxidation product-mediated inhibition of endothelial NO generation. Am J Physiol Cell Physiol 293: C1679–C1686. 17881609 10.1152/ajpcell.00224.2007

[46] Tan B, Jiang DJ, Huang H, Jia SJ, Jiang JL, et al (2007) Taurine protects against low-density lipoprotein-induced endothelial dysfunction by the DDAH/ADMA pathway. Vascul Pharmacol 46: 338–345. 17293168 10.1016/j.vph.2006.11.006

[47] Palm F, Onozato ML, Luo Z, Wilcox CS (2007) Dimethylarginine dimethylaminohydrolase (DDAH): expression, regulation, and function in the cardiovascular and renal systems. Am J Physiol Heart Circ Physiol 293: H3227–H3245. 17933965 10.1152/ajpheart.00998.2007

[48] Marliss EB, Chevalier S, Gougeon R, Morais JA, Lamarche M, et al (2006) Elevations of plasma methylarginines in obesity and ageing are related to insulin sensitivity and rates of protein turnover. Diabetologia 49: 351–359. 16369774 10.1007/s00125-005-0066-6

[49] Schutte AE, Schutte R, Huisman HW, van Rooyen JM, Fourie CM, et al (2010) Dimethylarginines: their vascular and metabolic roles in Africans and Caucasians. Eur J Endocrinol 162: 525–533. 19996198 10.1530/EJE-09-0865

[50] Zsuga J, Torok J, Magyar MT, Valikovics A, Gesztelyi R, et al (2007) Dimethylarginines at the crossroad of insulin resistance and atherosclerosis. Metabolism 56: 394–399. 17292729 10.1016/j.metabol.2006.10.023

[51] Simmons WW, Closs EI, Cunningham JM, Smith TW, Kelly RA (1996) Cytokines and insulin induce cationic amino acid transporter (CAT) expression in cardiac myocytes. Regulation of L-arginine transport and no production by CAT-1, CAT-2A, and CAT-2B. J Biol Chem 271: 11694–11702. 8662674 10.1074/jbc.271.20.11694

[52] Koc F, Tokac M, Erdem S, Kaya C, Unlu A, et al (2010) Serum asymmetric dimethylarginine levels in normotensive obese individuals. Med Sci Monit 16: CR536–CR539. 20980957

[53] Deneva-Koycheva TI, Vladimirova-Kitova LG, Angelova EA, Tsvetkova TZ (2011) Plasma asymmetric dimethylarginine levels in healthy people. Folia Med (Plovdiv ) 53: 28–33. 10.2478/v10153-010-0024-z 21644402

[54] Serg M, Kampus P, Kals J, Zagura M, Muda P, et al (2011) Association between asymmetric dimethylarginine and indices of vascular function in patients with essential hypertension. Blood Press 20: 111–116. 21142418 10.3109/08037051.2010.533821

[55] Kelly AS, Gonzalez-Campoy JM, Rudser KD, Katz H, Metzig AM, et al (2012) Carvedilol-lisinopril combination therapy and endothelial function in obese individuals with hypertension. J Clin Hypertens (Greenwich ) 14: 85–91. 22277140 10.1111/j.1751-7176.2011.00569.x PMC8108929

[56] Kandavar R, Higashi Y, Chen W, Blackstock C, Vaughn C, et al (2011) The effect of nebivolol versus metoprolol succinate extended release on asymmetric dimethylarginine in hypertension. J Am Soc Hypertens 5: 161–165. 21251896 10.1016/j.jash.2010.11.003 PMC3141281

[57] Pasini AF, Garbin U, Stranieri C, Boccioletti V, Mozzini C, et al (2008) Nebivolol treatment reduces serum levels of asymmetric dimethylarginine and improves endothelial dysfunction in essential hypertensive patients. Am J Hypertens 21: 1251–1257. 18772860 10.1038/ajh.2008.260

[58] Can A, Bekpinar S, Gurdol F, Tutuncu Y, Unlucerci Y, et al (2011) Dimethylarginines in patients with type 2 diabetes mellitus: relation with the glycaemic control. Diabetes Res Clin Pract 94: e61–e64. 21889812 10.1016/j.diabres.2011.08.008

[59] Abdi H (2007) The Bonferroni and Sidak corrections for multiple comparisons. In: Salkind NJ, editor. Encyclopedia of Measurement and Statistics. Thousand Oaks, CA: Sage. 103–107.

