# Supplementary figures and images for: Transsulfuration Pathway Thiols and Methylated Arginines: The Hunter Community Study
Source: PLoS One. 2013 Jan 24;8(1):e54870. doi: 10.1371/journal.pone.0054870 (PMC3554694; doi:10.1371/journal.pone.0054870)

## Slide 1
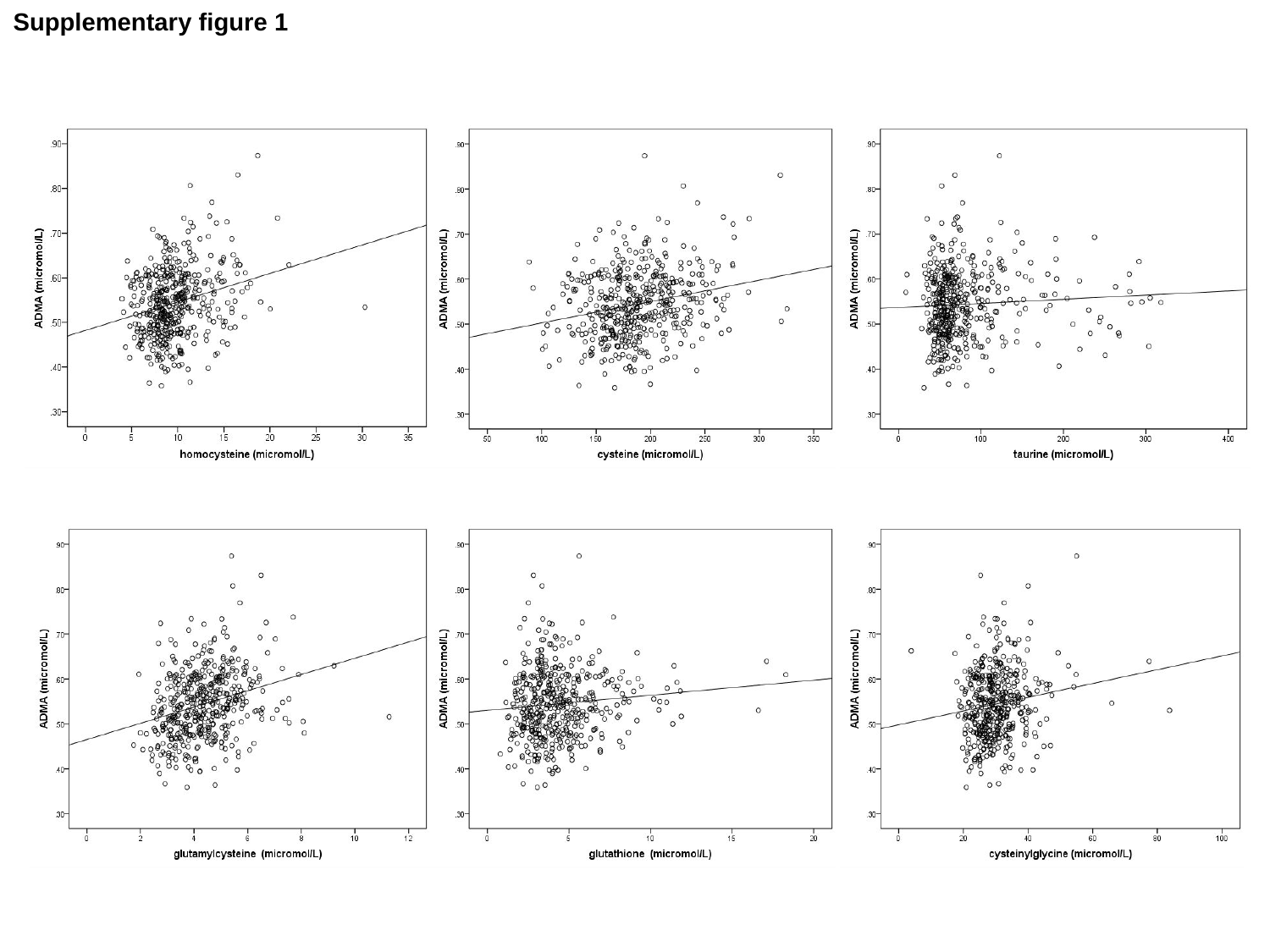

Supplementary figure 1

Supplement: Figure S1 — Scatter plots between individual serum thiols and ADMA concentrations. (PPTX) [file pone.0054870.s001.pptx]

## Slide 1
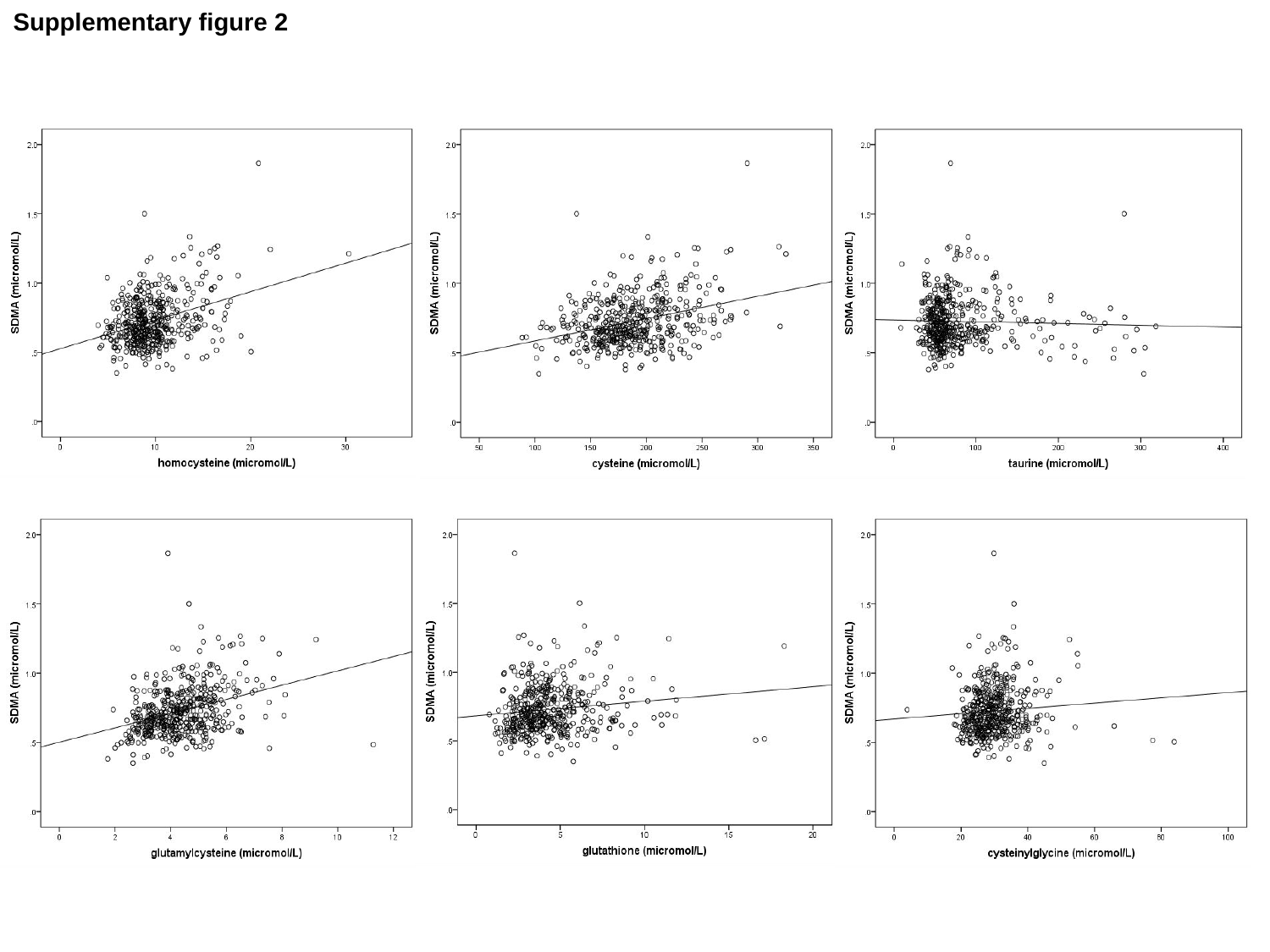

Supplementary figure 2

Supplement: Figure S2 — Scatter plots between individual serum thiols and SDMA concentrations. (PPTX) [file pone.0054870.s002.pptx]
